# Supplementary material for: Impact of Proton Pump Inhibitor Use on Progression-Free and Overall Survival in Cancer Patients Undergoing Immune Checkpoint Inhibitor Therapy: A Systematic Review and Meta-Analysis of Recent Studies
Source: Cancers (Basel). 2025 Jul 3;17(13):2228. doi: 10.3390/cancers17132228 (PMC12248532; doi:10.3390/cancers17132228)
Supplement: Supplementary file 1 [file cancers-17-02228-s001.zip › Supplementary File S2.pdf]

Supplementary File S2

Justification for the temporal scope restriction of the meta-analysis: a contiguity-based meta-analytical approach

The decision to limit the temporal range of this meta-analysis to studies published between November 2022 and January 2025 was driven by both methodological and scientific considerations. Prior meta-analyses covered available evidence up to November 2022 [the studies correspond to the references no. 16-22 of the manuscript]. Rather than diluting newly published evidence within a broader historical dataset, our objective was to perform a focused and contemporaneous synthesis to determine whether recent findings confirm, refine, or challenge the previously reported association between proton pump inhibitor (PPI) use and poorer survival outcomes in patients treated with immune checkpoint inhibitors (ICIs).

Although the literature search within the selected time frame was conducted systematically, we adopted a contiguity-based meta-analytical approach, in which the temporal window of the present study begins immediately after the endpoint of the preceding meta-analysis. This strategy ensures analytical continuity while allowing for the isolated evaluation of newly emerging evidence.

Several methodological advantages justify this choice:

1. By isolating studies published after November 2022, we avoid conflating the newly emerging evidence with earlier data, which could obscure temporal signals or evolving patterns of association.
2. The interaction between PPIs and ICI efficacy is a rapidly evolving field, fueled by growing interest in the immunomodulatory role of the gut microbiome. The identification of seven eligible studies in just over two years underscores the momentum of ongoing research in this area.
3. Standalone synthesis of post-2022 data enables the detection of temporal trends and methodological advancements (including refined multivariate adjustments, prospective study designs, biomarker-based stratification, etc.) that may not have been consistently applied in earlier work.
4. More recent studies are more likely to reflect updated clinical practices, including the introduction of newer ICIs, combinatorial immunotherapy regimens, and evolving supportive care protocols, thus enhancing the relevance of the findings to contemporary clinical decision-making.

This contiguity-based design aligns with principles of evidence updating and ensures that the current meta-analysis provides a timely, precise, and clinically relevant appraisal of the most recent literature.
