# Supplementary material for: Impact of Proton Pump Inhibitor Use on Progression-Free and Overall Survival in Cancer Patients Undergoing Immune Checkpoint Inhibitor Therapy: A Systematic Review and Meta-Analysis of Recent Studies
Source: Cancers (Basel). 2025 Jul 3;17(13):2228. doi: 10.3390/cancers17132228 (PMC12248532; doi:10.3390/cancers17132228)
Supplement: Supplementary file 1 [file cancers-17-02228-s001.zip › Supplementary File S3.pdf]

Supplementary File S3

Methodological aspects and outputs of the quality assessment scales employed in the study.

**Aspect evaluated**	**MINORS**	**NOS**
Study design	Explicit criteria for assessing non-randomized studies	Differentiates between cohort and case-control studies
Control group	Presence and appropriateness of a control group	Assesses the selection and comparability of the control group
Baseline comparability	Evaluates baseline comparability between groups	Examines comparability based on characteristics and confounders
Patient selection	Clear description of patient selection process	Criteria for patient selection and representativeness of the sample
Endpoint assessment	Adequate reporting and evaluation of study endpoints	Assess the outcome measurement and ascertainment of outcomes
Statistical analysis	Considers statistical analysis and power calculation	Considers statistical methods, appropriateness, and power
Loss to follow-Up	Addresses and accounts for loss to follow-up	Considers loss to follow-up and adequacy of follow-up duration
Confounding factors	Assesses the control of confounding factors	Addresses confounding factors and control measures
Interventions/Exposures	Evaluates the description and appropriateness	Examines exposure/intervention definition and measurement
Quality of reporting	Considers the overall quality of reporting	Examines the clarity and completeness of reporting
Score range (Min - Max)	0 - 16	0 - 9 (for cohort or case-control studies), 0 - 10 (for cohort studies)
Main aspect described	Assesses the overall methodological quality of non-randomized studies	Focuses on selection, comparability, and outcome assessment for observational studies
